# Conservation and diversification of small RNA pathways within flatworms

**DOI:** 10.1186/s12862-017-1061-5

**Published:** 2017-09-11

**Authors:** Santiago Fontenla, Gabriel Rinaldi, Pablo Smircich, Jose F. Tort

**Affiliations:** 1 Departamento de Genética, Facultad de Medicina, Universidad de la República (UDELAR), Gral. Flores 2125, CP11800 Montevideo, MVD Uruguay; 2 Parasite Genomics, Wellcome Trust Sanger Institute, Wellcome Genome Campus, Hinxton, Cambridge, CB10 1SA UK; 3 Laboratorio de Interacciones Moleculares, Facultad de Ciencias, Universidad de la República (UdelaR), Montevideo, Uruguay

**Keywords:** Flatworms, Small RNA pathways, miRNA, RNAi, Dicer, Argonaute

## Abstract

**Background:**

Small non-coding RNAs, including miRNAs, and gene silencing mediated by RNA interference have been described in free-living and parasitic lineages of flatworms, but only few key factors of the small RNA pathways have been exhaustively investigated in a limited number of species. The availability of flatworm draft genomes and predicted proteomes allowed us to perform an extended survey of the genes involved in small non-coding RNA pathways in this phylum.

**Results:**

Overall, findings show that the small non-coding RNA pathways are conserved in all the analyzed flatworm linages; however notable peculiarities were identified. While Piwi genes are amplified in free-living worms they are completely absent in all parasitic species. Remarkably all flatworms share a specific Argonaute family (FL-Ago) that has been independently amplified in different lineages. Other key factors such as Dicer are also duplicated, with Dicer-2 showing structural differences between trematodes, cestodes and free-living flatworms. Similarly, a very divergent GW182 Argonaute interacting protein was identified in all flatworm linages. Contrasting to this, genes involved in the amplification of the RNAi interfering signal were detected only in the ancestral free living species *Macrostomum lignano*. We here described all the putative small RNA pathways present in both free living and parasitic flatworm lineages.

**Conclusion:**

These findings highlight innovations specifically evolved in platyhelminths presumably associated with novel mechanisms of gene expression regulation mediated by small RNA pathways that differ to what has been classically described in model organisms. Understanding these phylum-specific innovations and the differences between free living and parasitic species might provide clues to adaptations to parasitism, and would be relevant for gene-silencing technology development for parasitic flatworms that infect hundreds of million people worldwide.

**Electronic supplementary material:**

The online version of this article (10.1186/s12862-017-1061-5) contains supplementary material, which is available to authorized users.

## Background

RNA interference (RNAi) is a reverse genetic tool that triggers post-transcriptional knock-down of a target mRNA by the introduction of complementary double-stranded RNA (dsRNA) molecules. It is almost the only available tool to study a gene function in helminths [1]. In platyhelminthes, while this technique has been routinely established in the study of gene function in planarians [2], is still poorly developed in parasitic species. In parasitic flatworms, RNAi techniques have been optimized in schistosome species [3], and it has been proven to be functional in other few trematode species like *Fasciola hepatica* [4–7], *Opisthorchis viverrini* [8], *Clonorchis sinensis* [9], *O. felineus* [10] and in the Cestodes *Moniezia expansa* [11], *Hymenolepis microstoma* [12], *Echinococcus granulosus* [13], *E. multilocularis* [14] and *Taenia crassiceps* [15]. While this suggest that the mechanism is fully functional, several caveats exist even in the more studied species, since inefficient or inconsistent transcript knockdown have been reported, highlighted by variable levels of gene silencing between parasite developmental stages and the unsuccessful silencing of target genes in *S. mansoni* [16–19] and *F. hepatica* [6]. Is still not clear if these variations could be related to difference in the accessibility of interfering molecules among developmental stages and/or variations in the RNAi silencing pathway trough the development. Therefore, measuring the expression levels of factors involved in RNAi in different stages would be relevant for the optimization of this functional approach. However, the lack of information regarding the genes involved in the RNAi pathway has been a limiting obstacle for this goal.

While the information on RNAi pathways in flatworms is fragmentary, several reports have detected the presence of microRNAs (miRNAs) in almost all the flatworm lineages [20–33]. MicroRNAs are relevant post-transcriptional regulators of gene expression conserved throughout all metazoa and plants [34]. Regulation mediated by miRNAs has been described in diverse biological processes including metabolism, cell development and differentiation, DNA methylation, chromatin modifications, neurological development, immune response, defense against viral infections, and cancer [35]. However, recent reports have shown a reduced miRNA complement in flatworms with significant sequence divergence in conserved families [23, 26], highlighting peculiarities of flatworm small RNA pathways.

The piRNA pathway is a small RNA mediated mechanism involved in the silencing of transposable elements in germline cells and hence, maintaining the genome stability [36]. While canonical 30–32 nt small RNAs typical of Piwi associated were detected in early studies in planarias [37–39], and later in *M. lignano* [22], they haven’t been found in the major parasitic flatworms. Consistent with this absence early studies seeking the PIWI-like protein in the model trematodes failed, leading to the question of how these organisms maintain their genome stability throughout generations [40].

Despite increasing evidence supporting the conservation of functional small RNA pathways in flatworms, they are still poorly described in most of the platyhelminth species. Reports comparing small RNA pathways among flatworms generally focus on the two main proteins, Dicer and Argonaute [40–42], while more comprehensive surveys of factors are limited to the analyses of one species at a time [22, 31, 43]. Given the scarce knowledge of the factors involved in small RNA pathways in platyhelminthes, we employed well-characterized biogenesis pathways of small non-coding RNAs in the model organism *Caenorhabditis elegans* to conduct a bioinformatic search in flatworms with available transcriptomic/genomic data.

## Results and discussion

The availability of 25 flatworm genome sequences, ranging from free living to parasitic monogenan, digenean and cestode lineages (Table 1) offer a good opportunity to compare and complete the still fragmentary knowledge of the small non-coding RNA pathways in platyhelminthes. Since the best described small non-coding RNA pathways are from *C. elegans,* we used a curated complete set of these factors as queries for comparisons (complete list in Additional file 1: Table S1).

**Table 1:** Analyzed species

CLASS	ORDER	FAMILY	SPECIES	GENOME SIZE	GENE COUNT	BIOPROJECT ID
RHABDITOPHORA	Macrostomida	Macrostomidae	*Macrostomum lignano* (Mli)	1040	60,534	*PRJNA284736*
	Tricladida	Dugesidae	*Schmidtea mediterranea* (Sme)	900	29,850	*PRJNA12585*
MONOGENEA	Monopisthocotylea	Gyrodactylidae	*Gyrodactylus salaris* (Gsa)	67	15,436	*PRJNA244375*
	Polyopisthocotylea	Polystomatidae	*Protopolystoma xenopodis* (Pxe)	617	37,906	*PRJEB1201*
TREMATODA	Opistorchida	Opistorchiidae	*Clonorchis sinensis* (Csi)	547	13,634	*PRJDA72781*
			*Opistorchis viverrini* (Ovi)	634	16,356	*PRJNA222628*
	Echinostomida	Echinostomatidae	*Echinostoma caproni* (Eca)	834	18,607	*PRJEB1207*
		Fasciolidae	*Fasciola hepatica* (FHO)	1139	15,739	*PRJNA179522*
			*Fasciola hepatica* (FHL)	1275	22,676	*PRJEB6687*
	Strigeidida	Schistosomatidae	*Trichobilharzia regenti* (Tre)	702	22,185	*PRJEB4662*
			*Schistosoma haematobium* (Sha)	385	13,073	*PRJNA78265*
			*Schistosoma japonicum* (Sja)	398	12,738	*PRJEA34885*
			*Schistosoma mansoni* (Sma)	362	10,772	*PRJEA36577*
CESTODA	Cyclophyllidea	Hymenolepididae	*Hymenolepis diminuta* (Hdi)	166	11,271	*PRJEB507*
			*Hymenolepis microstoma* (Hmi)	141	12,368	*PRJEB124*
			*Hymenolepis nana* (Hna)	163	13,777	*PRJEB508*
		Taeniidae	*Echinococcus granulosus* (Egr)	114	10,245	*PRJEB121*
			*Echinococcus multilocularis* (Emu)	114	10,663	*PRJEB122*
			*Taenia asiatica* (Tas)	136	10,331	*PRJEB532*
			*Taenia solium* (Tso)	122	12,481	*PRJNA170813*
			*Hydatigera taeniaeformis* (Hta)	104	11,649	*PRJEB534*
		Mesocestoididae	*Mesocestoides corti* (Mco)	117	10,614	*PRJEB510*
	Diphyllobotridea	Diphyllobothriidae	*Diphyllobothrium latum* (Dla)	531	19,966	*PRJEB1206*
			*Schistocephalus solidus* (Sso)	539	20,228	*PRJEB527*
			*Spirometra erinaceieuropaei* (Ser)	1259	39,557	*PRJEB1202*

Species abbreviation are indicated between brackets. Genome sizes (in Mb)

We observed that while the core of the pathways are generally conserved, several factors are absent in flatworms and/or have diverged to a degree that are unrecognizable on the primary sequence level, as described in the following sections. Is worth to mention here that genome annotation quality varies between different flatworm species, and for that reason we did not rely only on predicted proteomes for the study, since this might lead to misreporting unannotated or fragmented models as gene losses or gains. To avoid this issue we also performed full genomic searches and corrected gene annotations based on these results, extending partial genes or fully annotating putative novel ones (see Methods).

### Core ribonuclease III factors are differentially distributed in flatworms

**Fig. 1:**
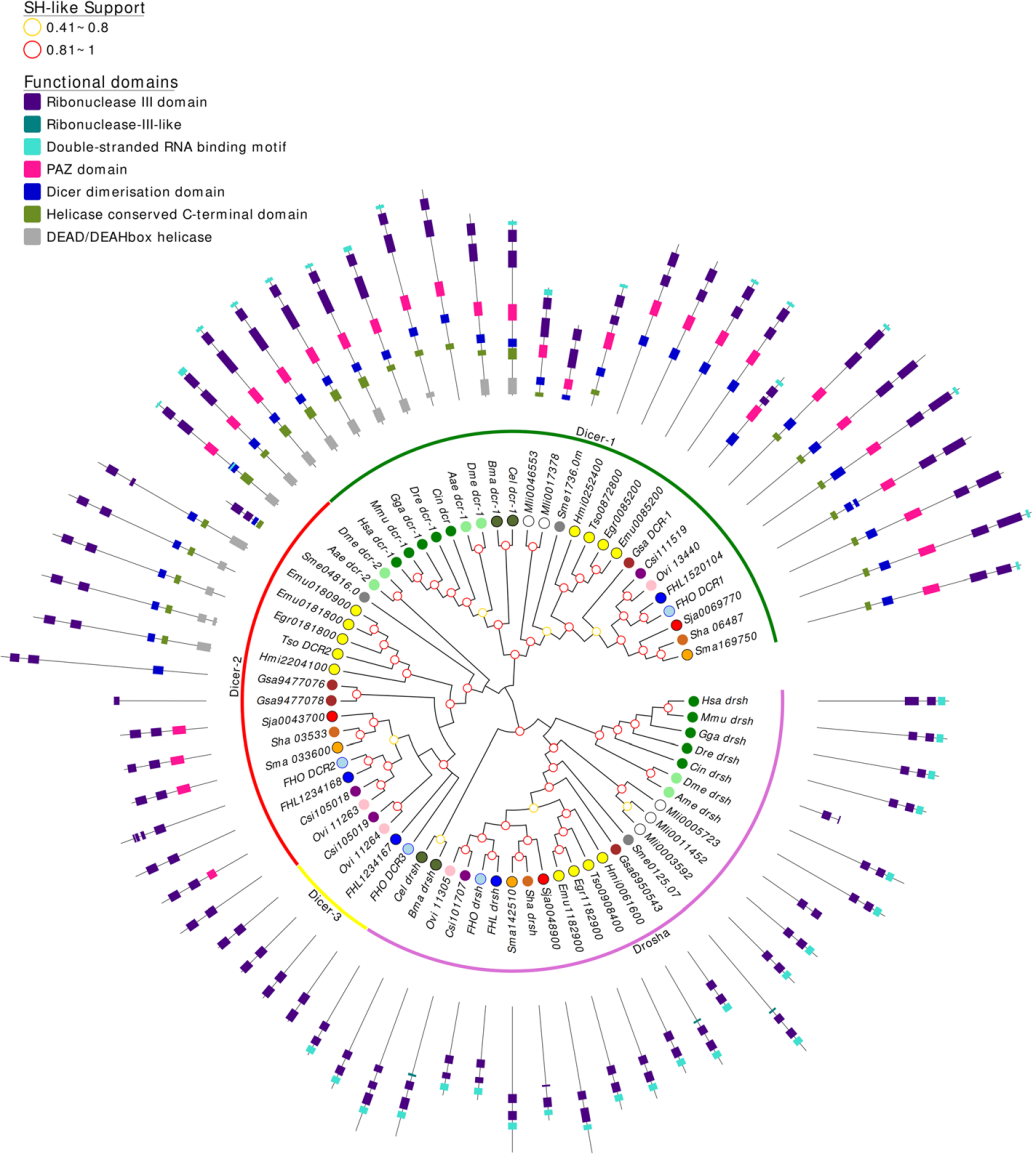
A maximum likelihood tree of Ribonuclease III subfamilies. SH-like approximate likelihood ratios are indicated, values below 0.4 are not shown. Conserved protein domains were predicted with HMMScan. Dcr-2 (red arch) has missing domains in trematodes compared to cestodes, monogeneans and planarians. Dicer-2 are duplicated in Fasciolidae and Opisthorchiidae but not in blood flukes, the genes product of duplication were named Dicer-3 (yellow arch). Species abbreviation are indicated in Table 1

The cleavage of long double stranded RNAs (dsRNA) by ribonuclease III enzymes is a key step in the biogenesis of small non-coding RNAs. Dicer (Dcr) belongs to this group of ribonucleases and process dsRNA into 22-23 nt RNAs with two-nucleotide 3′ overhangs that are recognized by Argonaute proteins [44]. Similarly, the nuclear protein Drosha (Drsh-1), involved in the generation of miRNA precursors, belongs to this group of ribonucleases. A complete conservation of *drsh-1* gene in all analyzed flatworms was observed, consistent with its central role in miRNA biogenesis (Fig. 1). While a single *drsh-1* gene has been described, two subfamilies of Dicer has been identified; a canonical Dcr-1 conserved in all metazoans, and a second subfamily, Dcr-2 present only in some species of invertebrates [41]. In *C. elegans*, there is an orthologue of Dcr-1, while in *D. melanogaster* there are orthologues of both subfamilies [41]. Expectedly, we found Dcr-1 conserved in all the flatworms analyzed, but also detected orthologues of Dcr-2 in all flatworms (Fig. 1). Interestingly, two copies of *dcr-2* genes were identified in all trematodes with the exception of the blood flukes (Additional file 1: Table S2). Dcr-2 and the second copy named Dcr-3, probably originated by an inverted duplication in a common ancestor of Fasciolidae and Opistorchidae (Additional file 2: Figure S1). To gain further insight into the organization of this family we analyzed the presence of functional domains. Flatworm Drsh-1 proteins showed two putative Ribonuclease III domains, a dsRNA-binding domain and N-termini rich in proline, serine and arginine residues (Fig. 1). On the other hand, variability in the organization of functional domains of Dcr-1 and Dcr-2 subfamilies has been reported [41]. The canonical Dcr-1 protein is characterized by an N-terminal DEAD/H-box helicase, a PAZ domain, two Ribonuclease III domains, and dsRNA-binding domain at the Carboxyl-end [44]. The predicted flatworm Dcr-1 proteins generally lack most amino terminal helicase domains conserving the remaining structure. While Dcr-2 proteins of insects present the conserved domain, this is drastically reduced in flatworms. In cestodes both the PAZ domain and the C-terminal dsRNA binding domain are absent. An even shorter Dcr-2 is present in trematodes, with only two RNAse III domains. A PAZ domain was also identified in schistosomes and in *C. sinensis*, suggesting the presence of highly divergent domains in the remaining trematodes. The presence of divergent Dcr-2 in flatworms, and Dcr-3 in Fasciolidae and Opistorchidae may indicate functional redundancy or different roles for both proteins. Available transcriptomic data from *S. mansoni* and *F. hepatica* shows that all these variants are expressed among different developmental stages; particularly, *F. hepatica dcr-3* is predominantly expressed in eggs (Additional file 2: Figure S1).

In *D. melanogaster* Dcr-1 and Dcr-2 proteins have evolved different roles, Dcr-1 processes pre-miRNA, i.e. miRNA precursor molecules, while Dcr-2 processes dsRNA in the RNAi pathway [45]. Dcr-1 would preferentially bind and process non-complementary stretches of dsRNA, characteristic of miRNAs, while Dcr-2 requires perfect complementarity between the dsRNA and target mRNA [46]. Due to the conservation of both subfamilies is tempting to hypothesize that a similar phenomenon may be occurring in flatworms, however this hypothesis awaits to be demonstrated.

### Parasitic flatworms have two groups of Argonaute, while planarians have three

Argonaute proteins are essential for gene regulatory mechanisms mediated by small RNAs. The Argonaute (Ago) genes have been classified into four paralogous groups: (1) Argonaute-like (Ago-like) proteins, similar to *Arabidopsis thaliana* Ago1; (2) Piwi-like proteins, closely related to *D. melanogaster* PIWI; (3) a specific group identified in *C. elegans* called group 3 [47, 48], reported to be conserved in nematodes [49]; and (4) a cluster of proteins flatworm-specific recently identified and named cluster 1 by Zheng [42] and cluster 4 by Skinner [40].

In agreement with previous reports, our exhaustive homology search among diverse flatworms showed multiple hits in all species ranging from 3 in cestodes to more than a dozen in free-living species. A more detailed phylogenetic analysis aided by structure determination allowed us to further classify these hits.

**Fig. 2:**
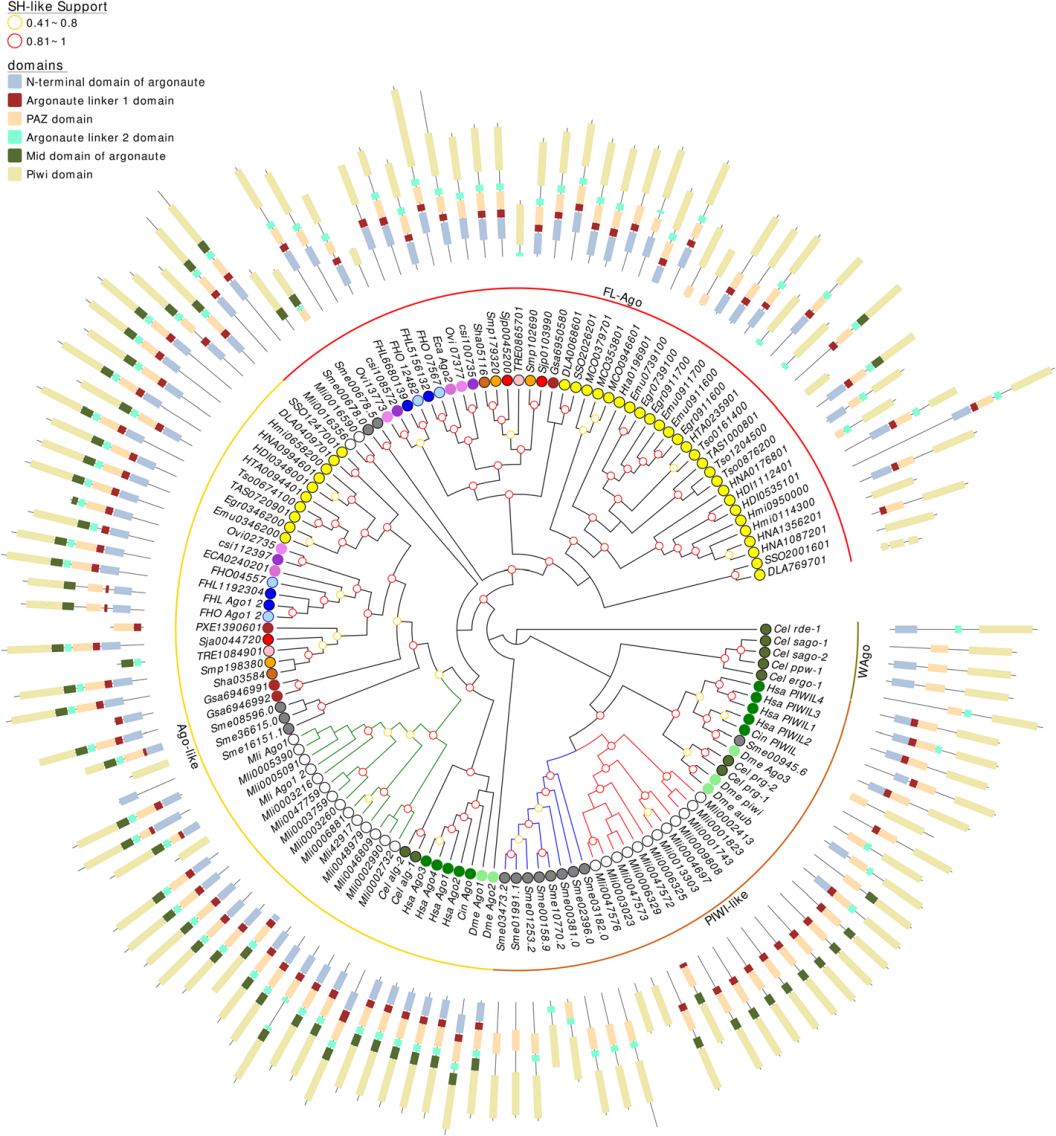
A maximum likelihood tree of Argonautes. SH-like approximate likelihood ratios are indicated, values below 0.4 are not shown. Conserved protein domains were predicted with HMMScan. Green branches indicate the expansion of canonical Ago-like (yellow arch) in *M. lignano*. Blue branches and red branches indicate of PIWI-like (brown arch) in *S. mediterranea* and *M. lignano*, respectively. FL-Agos are indicated with the red arch. Species abbreviation are indicated in Table 1

We found single orthologues of the canonical Ago subfamiliy in most parasitic flatworms, and an expansion in free living species. While *S. mediterranea* showed tree paralogues, in *M. lignano* thirteen paralogous sequences were detected (Fig. 2, Additional file 1: Table S3); this massive amplification seem consistent with the suspected tetraploidy of *M. lignano* [50].

Besides this, the monogenan *G. salaris* showed two inverted paralogous genes adjacent on the same contig suggest a recent duplication. Also in *F. hepatica* two paralogues were detected by genomic scanning, one of them unannotated in both genome assemblies available (Fig. 2, Additional file 1: Table S3). However, this novel unannotated gene has an unusual gene structure and is poorly expressed, suggesting that it might correspond to a pseudogene.

Interestingly, the domain architecture of these Ago-like proteins is very conserved from vertebrate to flatworms, i.e. an N-terminal domain followed by a linker 1 domain, the PAZ domain, which is important for the small RNA association along with the PIWI domain [48], a linker 2 domain, followed by the mid domain and PIWI, the catalytic domain (Fig. 2).

The PIWI-like proteins are core factors of the piRNA pathway involved in transposable elements (TE) silencing, and hence, maintaining the genome stability in germline cells [36]. This group was first reported in *S. mediterranea* [37, 38] and *Dugesia japonica* [39], and more recently in *M. lignano* [22]. According to these early observations, we detected Piwi-like genes only in turbellarians, with a notable amplification in the genomes of both free-living species (Fig. 2, Additional file 1: Table S3). Again, the distribution of copies in *M. lignano* show amplification rounds consistent with tetraploidy. Surprisingly, structure of PIWI-like proteins from free-living flatworms showed some variations, while most *S. mediterranea* PIWIs have the linker 2 domain besides the PAZ and PIWI domains, *M. lignano*‘s have the linker 1 and mid domains instead (Fig. 2). The apparently loss of PIWI-like genes during the evolution of parasitic platyhelminthes led to the question of how these organisms maintain their genome stability throughout generations [40].

While the presence of flatworm-specific Agos have been previously noticed in model species [40, 42], we now found that this group of flatworm-specific Agos (named FL-Ago by us), is actually amplified, being represented by two or three paralogous genes identified in each of the parasitic species (Fig. 2, Additional file 1: Table S3). The phylogenetic analysis suggests that independent duplications within the FL-Ago have occurred in both trematodes and cestodes. We used HMMScan and MEME to inspect FL-Agos domains finding that in most of them the mid domain was missing. The mid domain intervenes in the anchor of the 5′ phosphate of siRNA or miRNA and contains a cap-binding region that is required for efficient regulation of translation [48]. Additionally, the transcriptomic data of *S. mansoni*, *F. hepatica* and *E. granulosus* showed that FL-Agos were being expressed in several stages (Additional file 3: Figure S2). Even though we can speculate that the function of FL-Agos might be relevant it remains unknown. However, Cai et al. [51] sequenced the population of small RNA associated to Ago2 of *S. japonicum*, detecting 19-22 nt endo-siRNA derived from TE. These results suggested that Sj.Ago2 might be a functional homologue of the *D. melanogaster* Ago2 in the short-interfering RNA (siRNA) pathway suppressing active TE in both the somatic and germline cells [40]. Considering that in all flatworm lineages different Ago proteins are amplified is tempting to speculate that they might represent diverse adaptations to cope with similar phenomena.

### Most proteins of the miRNA pathway are conserved in flatworms

**Fig. 3:**
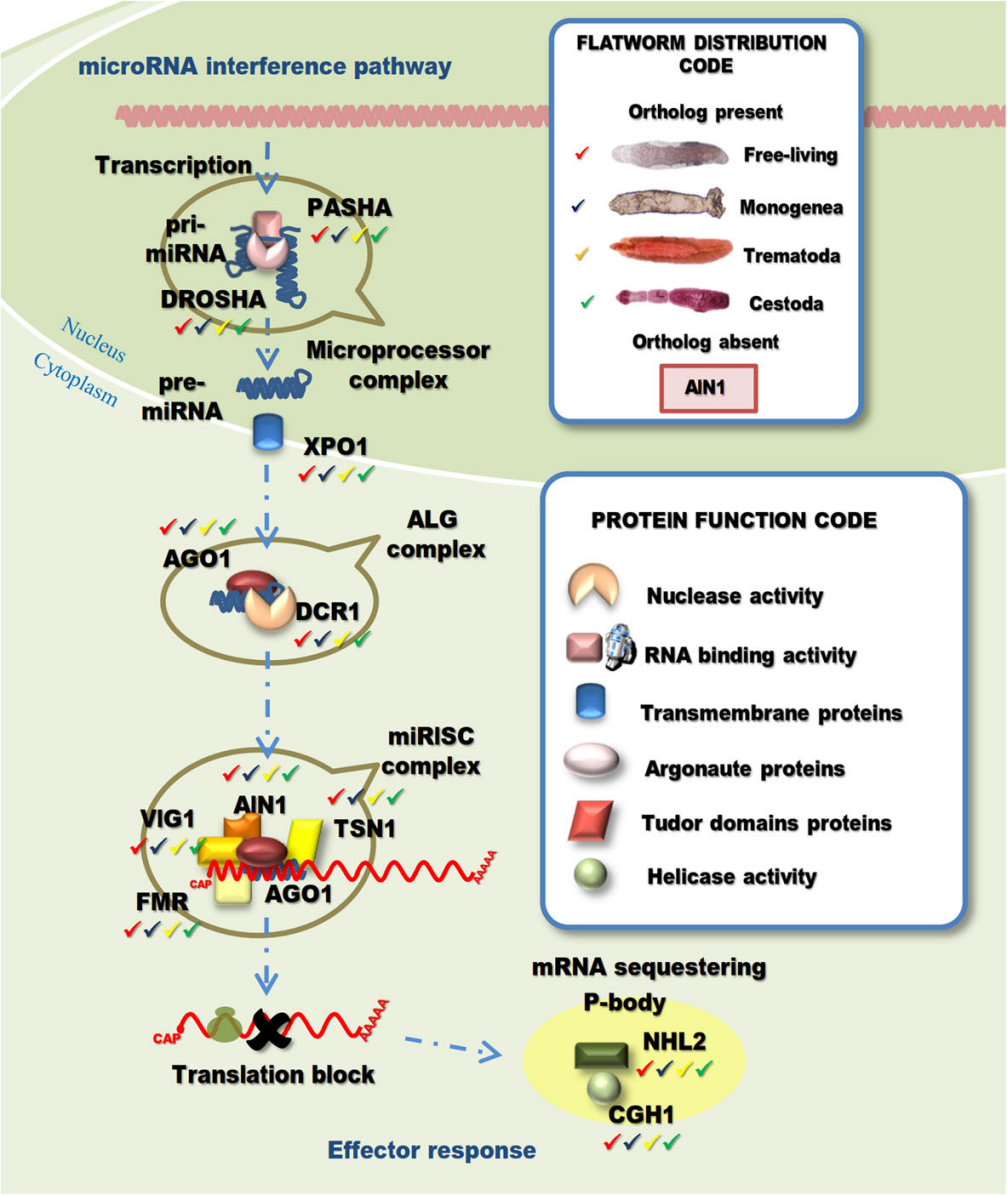
MicroRNA pathway genes detected in flatworms. Pri-microRNA is transcribed in the nucleus by RNA polymerase II and processed into pre-microRNA by Pasha and Drosha (the microprocessor complex). Pre-miRNA is exported to the cytoplasm by the transmembrane protein Xpo1 where is processed by Dicer into a 21 ~ nucleotides dsRNA. Ago1/2 removes the passenger strand from the mature miRNA. The miRNA with Ago1 and other factors associated to the miRISC complex will anneal the target mRNA with 100% complementarity to the seed region (nucleotides 2–8 of miRNA) blocking the translation and sequestering the mRNA to the P-body. Factors involved in the microRNA pathway with homologous genes detected in flatworms are indicated (“Flatworm Distribution Code” box). A ‘shape’ code was used to indicate predicted function of factors (“Protein Function Code” box)

Pri-microRNAs are transcribed by RNApol II and processed into 60–100 nucleotides long pre-miRNA by the RNase III Drosha and its partner Pasha in a complex named microprocessor [52]. We confirmed that Drosha and Pasha are conserved in all the flatworm analyzed (Fig. 3, Additional file 1: Table S4). In addition, we identified orthologous genes of the transmembrane channel Xpo-1 that transports pre-miRNAs into the cytoplasm in *C. elegans* [53]. In vertebrates and flies Exp-5, that connects the nucleoplasm with the cytoplasm, is involved in the miRNA pathway; however, in nematodes no Exp-5 orthologue has been identified and it has been hypothesized that Xpo-1 replaces its function [53, 54]. An orthologue of Exp-5 has already been described in *S. mansoni* [43] and we detected orthologues in other flatworms by using SmExp-5 as query in tBLASTn search (Additional file 1: Table S4). In *C. elegans*, Dcr-1 further process pre-miRNAs into a ~ 22 nt RNA duplex [55]. In *D. melanogaster*, however, Dcr-1 requires Loquacious (Loqs), a dsRNA-binding protein, for pre-miRNA processing [45]. Loqs orthologues were identified in most of flatworm species analyzed (Additional file 1: Table S4), suggesting that this protein may be relevant for RNAi processing in flatworms.

Argonaute gene products (ALG-1 or ALG-2 in *C.elegans*, Ago1 in *D. melanogaster*) recognize short RNA duplexes, cleaving the passenger strand from the mature miRNA. The Ago protein in tandem with the miRNA and other associated factors form the miRNA Induced Silencing Complex (miRISC) that lead to the degradation of target mRNAs [55].

### GW182 Is conserved in free living *M. lignano* and divergent in parasitic parasites

**Fig. 4:**
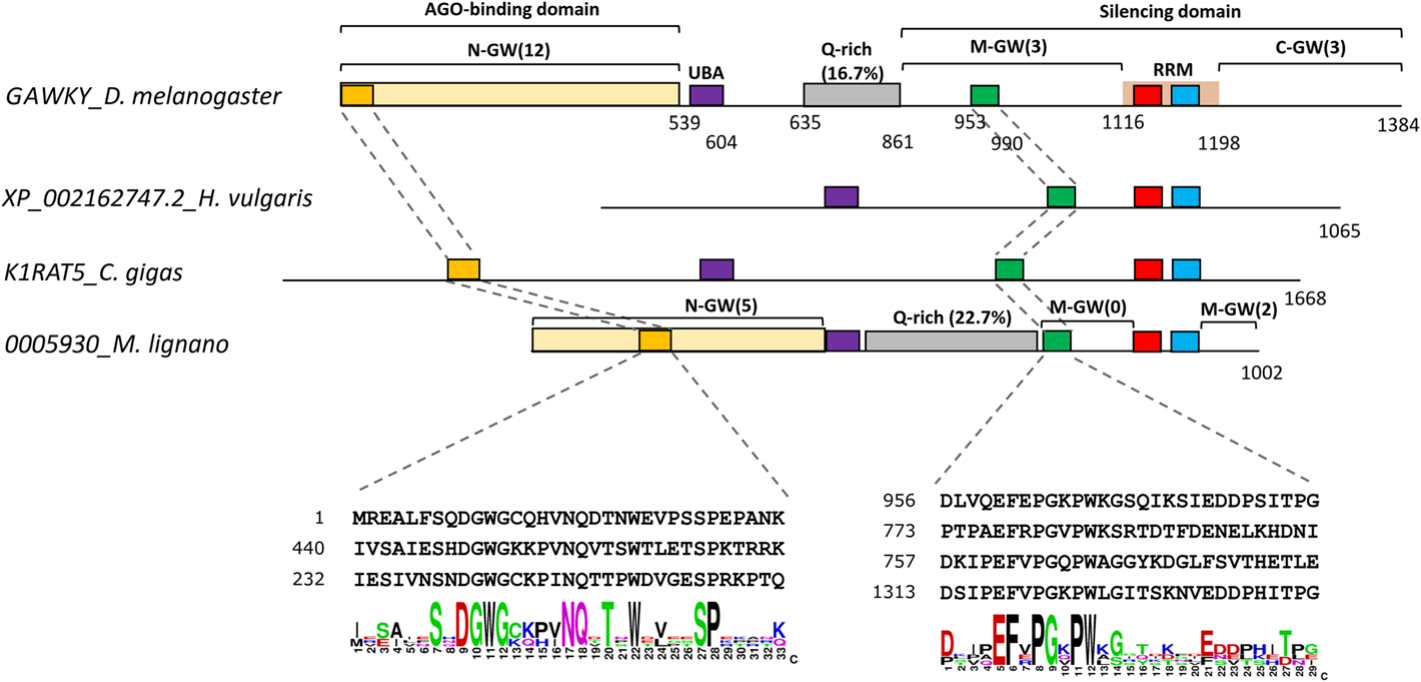
Conserved regions in *M. lignano* GW182 family member. GW182 interacts with Ago and ‘label’ mRNAs for decay. A GW182 family member has two protein domains; a central Ubiquitin domain (UBA), and a RNA recognition motif (RRM), and unstructured regions in between domains. The N-termini region (N-GW) binds to Ago, and the middle (M-GW) and carboxi-terminal region (C-GW) mediate the decay. Each of these regions consists on a variable number of GW repeats (number between brackets). Additionally, there is a low complex region rich in glutamine (Q-rich) between UBA domain and M-GW region. There are two highly conserved sequences in N-GW (yellow box) and M-GW (green box) regions that we found to be conserved in *M. lignano* sequence

GW182 is one of the most critical miRISC factors. In *D. melanogaster*, Gawky (GW182 orthologue) contributes to translation repression and ‘label’ the target mRNA for decay via deadenylation and decapping [56]. Moreover, knock-down of GW182 resulted in the suppression of mRNA silencing mediated by miRNA with no effect on the expression levels of corresponding miRNAs or Argonaute protein [57]. GW182 family members share a common domain characterized by a central ubiquitin associated-like domain (UBA) and C-terminal RNA recognition motif (RRM). These domains are embedded in regions predicted to be unstructured that include three blocks of glycine-tryptophan repeats (the N-, middle- and C-terminal GW-repeats), and a glutamine-rich (Q rich) region located between the UBA and the RRM domains, that are difficult to retrieve by simple homology searches (Fig. 4) (reviewed at [57]). On the other hand, two proteins (AIN-1 and AIN-2) with a low number of GW-repeats but no Q-rich region or UBA and RRM domains has been described in *C. elegans*. This lack of common domain architecture suggests that AIN-1 and AIN-2 may not be members of the GW182 protein family, but rather represent functional analogs [57].

AIN-1 and AIN-2 from *C. elegans*, Gawky from *D. melanogaster,* and a GW182 family member recently reported in *Crassostrea gigas* [58], were employed as queries to interrogate the flatworm genomes in Wormbase parasite. Hits were only identified in the genome of *M. lignano* using the *C. gigas* sequence, probably due to closer evolutionary distance between these species. The *M. lignano* sequence was predicted to contain UBA and RRM domains like the GW182 family member. Despite the unstructured nature of GW182 family members, outside UBA and RRM domains there are two short but well conserved motifs in the Ago-binding domain and the silencing domain (Fig. 4) [57]. Using MEME suite, motifs were found to be partially conserved in *M. lignano* sequence (Fig. 4). In addition, we found a Q-rich region with 22.7% of glutamine residues between the UBA domain and the conserved motif of silencing domain as previously described for the Gawky sequence (Fig. 4). This suggests that the free living *M. lignano* has at least one GW182 family member. Next, we used this *M. lignano* putative GW182 as bait to search by tBLASTn in the genomes of other flatworms, finding several hits with different confidence, mainly in proteins with unknown function (Additional file 1: Table S4). MEME search on these putative GW182 orthologues identified the presence of dispersed sequence conservation (Additional file 4: Figure S3). Experimental validation of the role of the putative GW182 is now needed to confirm its essentiality in miRNA-driven gene silencing and the interaction with the miRISC complex in flatworms.

### Other miRISC factors are moderately conserved in flatworms

Besides the factors analysed above, other proteins have been described in the miRISC, mainly RNA binding proteins [55, 59, 60]. Homologues genes of TSN-1 and VIG-1 were identified in almost all flatworms (Additional file 1: Table S4). We confirmed the presence of an orthologue to Fragile X Retardation factor (FXR) involved in miRNA silencing in mammals previously reported in *S. mansoni* [43]. On the other hand, no RNA binding protein GLD-1 orthologues were identified in our survey; however, we detected orthologue proteins for ASD-2, a paralog of GLD-1 in *C. elegans*, which might be a functional homolog in flatworms.

### The exoRNA interference is functional in flatworms

**Fig. 5:**
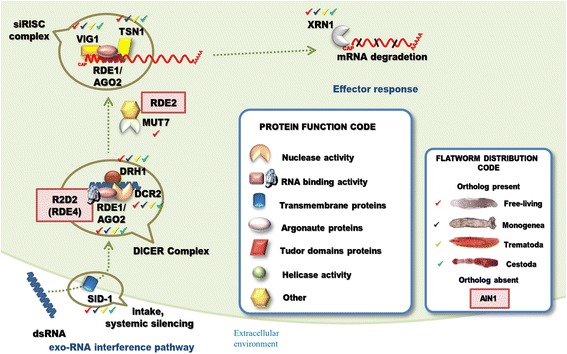
Exogenous RNA interference pathway genes detected in flatworms. An exogenous double-stranded RNA is internalized by transmembrane channels SID-1. In the cytoplasm the dsRNA is recognized by RDE4 (an orthologous of R2D2 described in *D. melanogaster*), this promotes the formation of the DICER complex that process the long dsRNA into 22 nucleotides long dsRNA. The Argonaute protein RDE1 (homologous to Ago2 in *D. melanogaster*) further process the dsRNA releasing matures siRNA that together with other factors form the effector complex siRISC. mRNAs targeted by siRISC are degraded, XRN1 has 5′-3′ exonuclease activity and intervenes in this process. Mut-7 and RDE-2 act downstream of RDE-1 and RDE-4 and are required for siRNA accumulation. Exogenous RNAi pathway factors with homologous genes detected in flatworms are indicated (“Flatworm Distribution Code” box). A shape code was used to indicate predicted function of factors (“Protein Function Code” box)

The RNAi pathway discovered at the dawn of the century in *C. elegans*, is now known as the exo-RNAi pathway (Fig. 5). The mechanism is triggered when a long dsRNA molecule is internalized by the cell by a transmembrane channel protein called SID-1 [61]. Other transmembrane coding genes paralogous to SID-1 in *C. elegans* have been described; CHUP-1, Y37H2C1 and C08A9.3. However, none of them seem to be involved in RNAi, CHUP-1 in particular, is expressed mainly in the intestine and the terminal bulb of the pharynx where is involved in the internalization of cholesterol [62]. Interestingly, in a pairwise distance matrix of aligned sequences we observed that platyhelminthes, insects and vertebrates SID-1 homologues (named Sid-like by [63]) are closely related to CHUP-1 than to SID-1. Thus, platyhelminthes, insects and vertebrates SID-like transporters might instead be orthologous of *C. elegans* CHUP-1 [63]. It has been recently reported that like *C. elegans* SID-1, vertebrates SID-like binds to dsRNA through its extracellular domain [64] suggesting that the role of SID-1 might be exerted by SID-like proteins in other organisms.

**Fig. 6:**
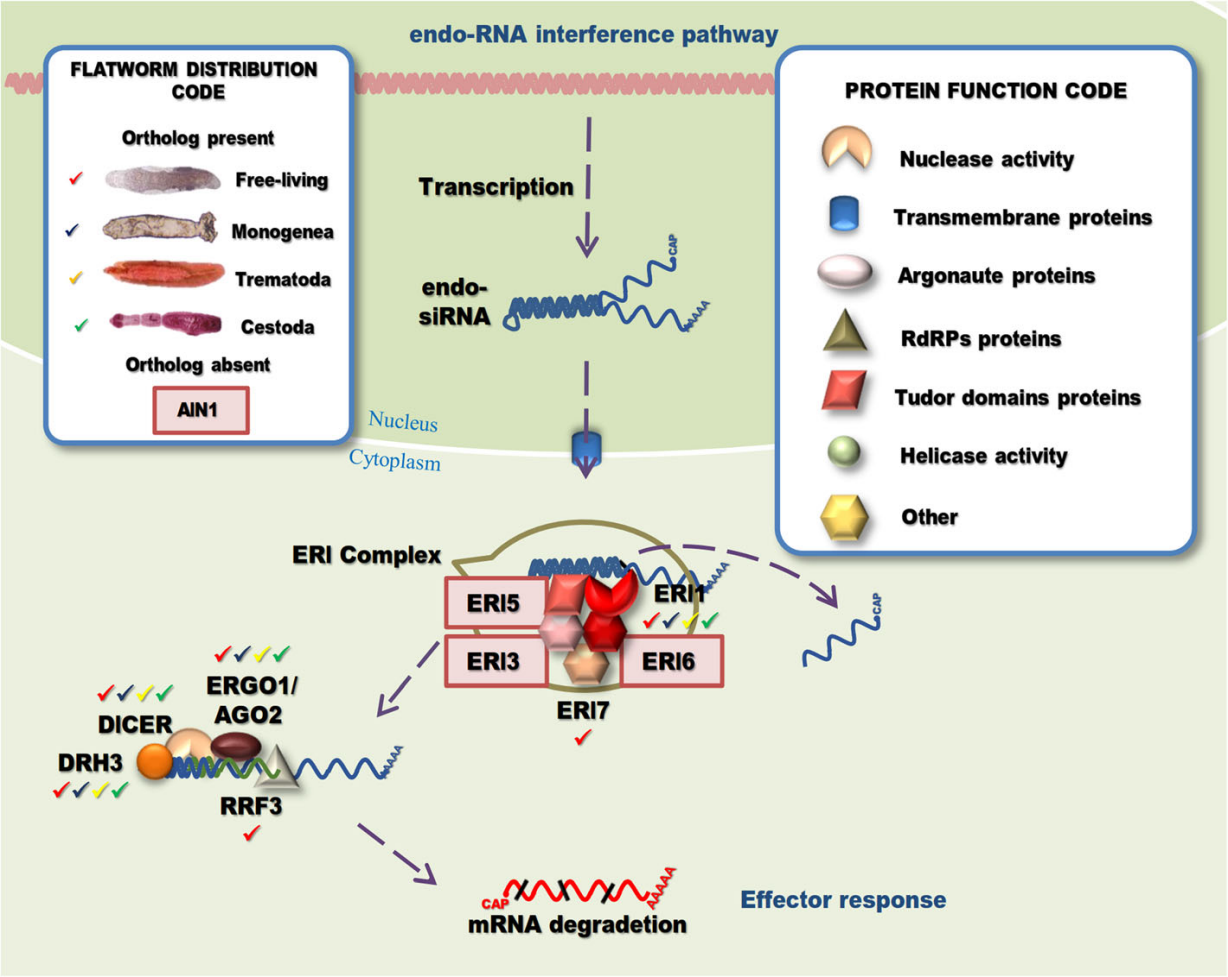
Endogenous RNA interference pathway genes detected in flatworms. The non-coding RNA (ncRNA) containing a dsRNA region is transcribed in the nucleus and processed in the cytoplasm by ERI complex. After binding, the ncRNA ERI-1 removes one of unpaired strands creating a hairpin structure suitable for priming the synthesis of long dsRNA by RRF-3. Dicer and DRH-3 then, cleave the long dsRNA, and ERGO-1 (homologous to Ago2 in *D. melanogaster*) loaded with siRNA triggers the target RNA degradation. Endogenous RNAi pathway factors with homologous genes detected in flatworms are indicated (“Flatworm Distribution Code” box). A ‘shape’ code was used to indicate predicted function of factors (“Protein Function Code” box)

**Fig. 7:**
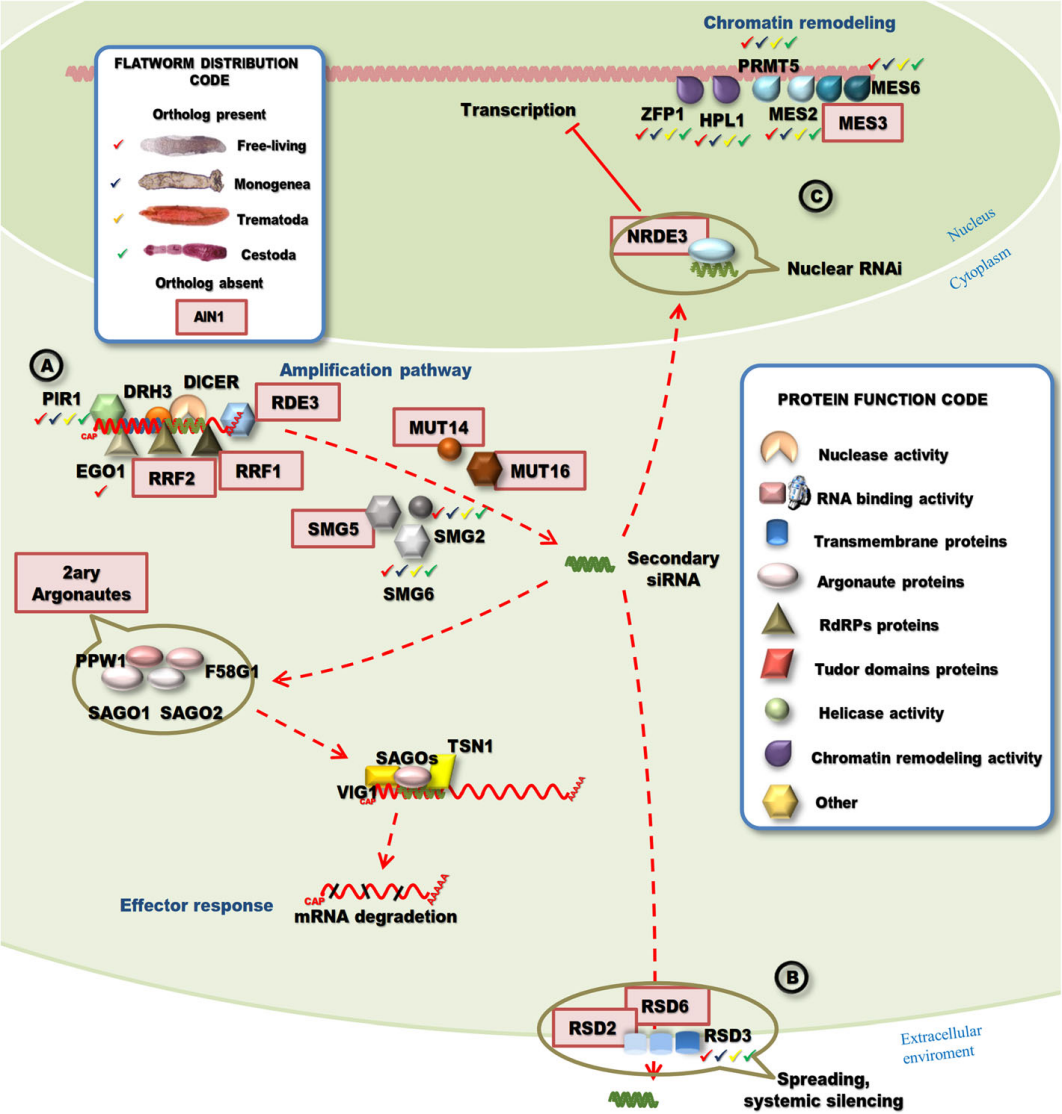
RNAi amplification, spreading and transcriptional gene silencing pathways. **a** Amplification pathway. RNA dependent RNA Polymerases (RdRP) employs the 3′ of the siRNA as primer to synthesis a new long dsRNA using the mRNA as template. PIR-1 and DRH-3 are required for Dicer to process long dsRNA, while RDE-3 intervenes in an intermediate step, stabilizing the product of the initial round of cleavage, allowing the amplification mediated by RdRPs. SMG genes, Mut-14 and Mut-16 act downstream of secondary siRNA generation. RNAi persists in the animals exposed to dsRNA for several days [5]. This persistence depends on SMG genes. The secondary siRNA with the Secondary Argonautes (SAGOs) produce a secondary siRISC complex. **b** Systemic spreading. The secondary siRNAs spread throughout other tissues inducing the systemic silencing of the target gene. **c** Co-transcriptional silencing and chromatin remodeling. A specialized Argonaute, NRDE-3, transports the secondary siRNA to the nucleus where produce co-transcriptional gene silencing and interacts with other factors to remodel the chromatin. Factors with homologous genes detected in flatworms are indicated (“Flatworm Distribution Code” box). A shape code was used to indicate predicted function of factors (“Protein Function Code” box)

**Fig. 8:**
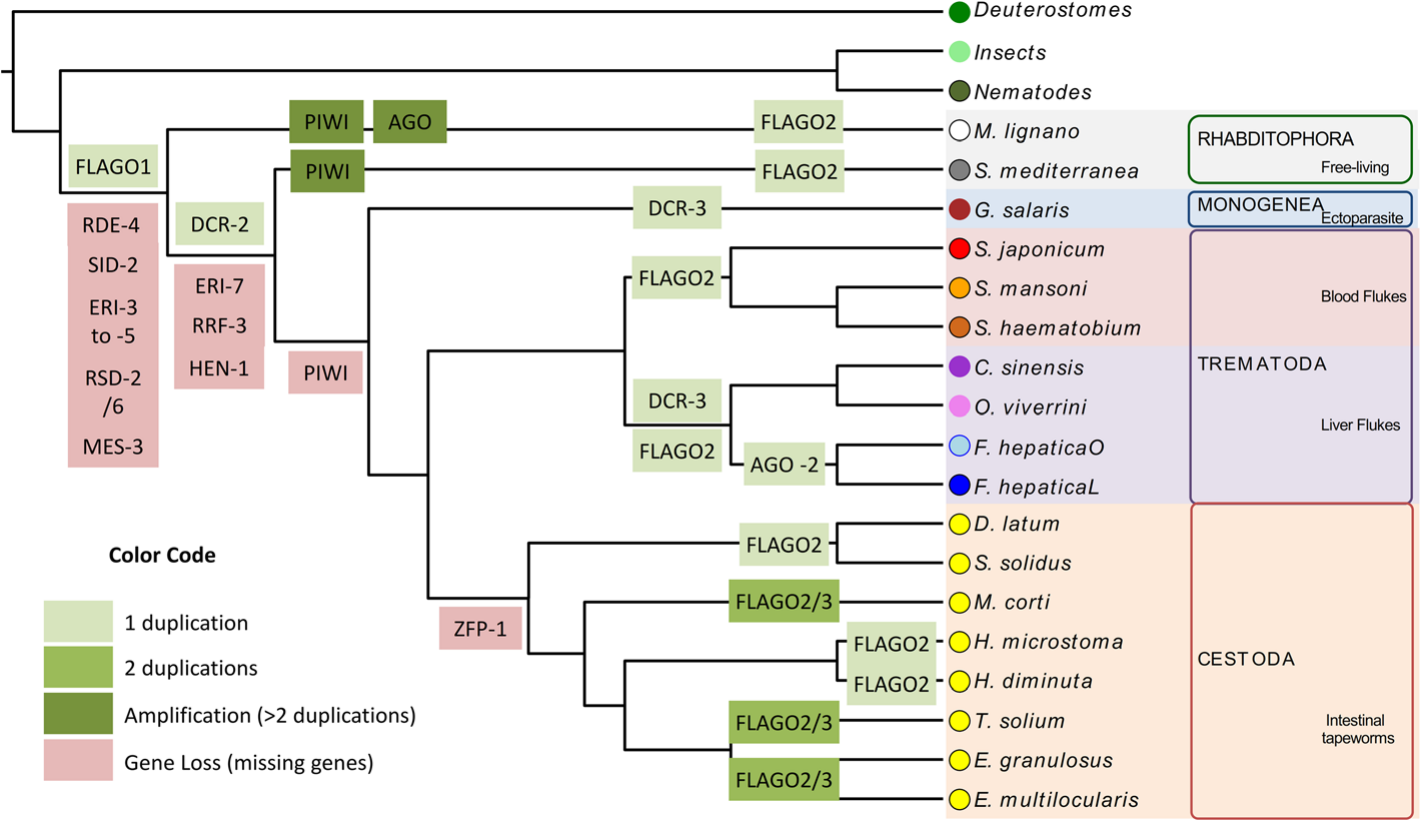
Summary of small RNA pathways gene gains and losses in the evolution of flatworms. A schematic representation of the phylogeny of flatworms indicating major gene duplications or gene losses

Other key transmembrane protein involved in dsRNA uptake in *C. elegans* is SID-2. Interestingly, SID-2 is required for RNAi delivered by dsRNA soaking or feeding (environmental RNAi), but not for systemic RNAi. The protein is mainly expressed in the apical intestinal membrane where in coordination with SID-1 internalizes the interfering molecules [65]. Even though RNAi by soaking has been proven to be functional in platyhelminthes [5, 9, 16], we did not detected orthologues of SID-2 in flatworms. Therefore, it is possible that the role of SID-2 has been substituted by other proteins that enable the preliminary uptake of dsRNA like SID-1 [65].

In *C. elegans*, the dsRNA is first recognized by the dsRNA-binding protein RDE-4 and presented to DCR for processing in the cytoplasm [66]. RDE-4 is conserved only in some nematode species [16, 49], hence, orthologues of R2D2, the functional homologous of RDE-4 reported in *D. melanogaster* [67]*,* were searched. No proteins similar to RDE-4 or R2D2 were identified in flatworms, an intriguing observation considering its critical role in the generation of siRNAs [68]. However, since RNAi has proven to be functional in several flatworms, an alternative pathway or other yet uncharacterized mediators might be operating in these organisms.

In addition to Dcr and Ago genes, we identified putative homologs of DRH-1, a DExH-box helicase protein associated to Dcr and XRN-1, a 5′-to-3′ exonuclase that degrades the mRNA targeted by the RISC complex (Fig. 5) (Additional file 1: Table S5).

No homologs of Mut-7 and RDE-2 in flatworms were detected; these factors are involved in a protein complex in *C. elegans*, required for downstream events in the RNAi not well understood [69]. In addition, Mut-7 orthologues were described in *D. melanogaster* and *H. sapiens* [70].Therefore, we used Nbr (the orthologue of Mut-7 in *D. melanogaster*) as query to search by tBLASTn on the flatworm genomes. Only *M. lignano* showed three genomic regions with high similarity (43.3% on average), two of them with annotated genes. These genes were used as bait to test the other flatworm genomes with no hits identified. Therefore, it is possible that Mut-7 homologs has been lost in the evolution of flatworms or a high evolution rate within the flatworm phylogeny made them undetectable by our method. RDE-2, on the other hand, has only been detected in *C. elegans* and other *Caenorhabditis* species [49, 71]. We can speculate that either the role of this complex is not crucial in flatworms or that there are other non-evolutionary related factors playing a similar role.

### The endo-RNAi pathway is reduced in parasitic flatworms

In *C. elegans*, in addition to exo-siRNA, a endo-RNAi pathway triggered by endogenous dsRNAs derived from overlapping genes, long hairpins, and TE in the germline, has been reported [72, 73]. The primary transcripts are processed by the Enhancer of RNA Interference (ERI) complex formed by Dcr-1, DRH-3, ERI-1, ERI-3, ERI-5, ERI-6, ERI-7 and RRF-3, a RNA-dependent RNA polymerase (RdRP) [73–75]. The suppression of the ERI factors increased the RNAi effect triggered by exogenous dsRNA [76, 77], suggesting that the exogenous and endogenous RNAi pathways shared common factors [74, 78].

In flatworms, we only detected the presence of ERI-1, a ribonuclease with an 3′-5′ exonuclease domain (Fig. 6). Interestingly, ERI-1 putative orthologues were lost in the teanid and hymenolepid cyclophilidean cestodes, while still present in *M. corti*. In addition, in the Diphyllobothriidae order only *D. latum* seem to have lost this gene (Additional file 1: Table S6). On the other hand, we detected homologue sequences of R02D3.8, a paralogous of ERI-1, in almost all platyhelminthes, including the species lacking an ERI-1 orthologue (Additional file 1: Table S6). It is possible that in cestode species lacking ERI-1 its function has been substituted by homologs of R02D3.8. In any case, experimental evidences are needed to prove this hypothesis. While we were unable to detect any other member of the ERI group in parasitic flatworms, putative orthologues of NTP hydrolase ERI-7, are found in the free living species *M. lignano* and the planarian *S. mediterranea.* As happens with other factors already described, *M. lignano*, has three predicted genes with high homology to RRF-3, i.e. the RdRP of the endo-RNAi, and like the ERI factors, it has been reported that the silencing of RRF-3 increased the effect of exo-RNAi [79].

### The RNAi amplification pathway is functional in *M. lignano*

In addition to the primary silencing, a pathway that amplifies the interfering signal has been reported in *C. elegans* [55]. RNA dependent RNA Polymerases (RdRP) that polymerase dsRNA using the target mRNA bonded to the siRNA as template are protagonists in this pathway. The amplification pathway is not conserved in metazoans, being absent in insects and mammals [70].

Among the thirteen *C.elegans* factors involved in the amplification pathway, we identified PIR-1, SMG-2 and SMG-6 conserved in flatworms, but no RDE-3 orthologues were detected. However, we did find homologous sequences to its paralogous gene GLD-2 (Fig. 7a, Additional file 1: Table S7).

In addition, four RdRPs were identified in *M. lignano,* one of them with high homology to EGO-1, which plays a critical role in the adult germline exo-RNAi pathway [80]. To detect low conserved homologs of RdRPs in other flatworms, we used *M. lignano* genes as queries in tBLASTn searches; however, no evident homologs were detected.

RdRPs are conserved in early branches of the tree of life, i.e. plants, fungi, yeast and nematodes [49, 81] Therefore, the possibility that *M. lignano* RdRPs derived from contaminants such as virus, fungi or yeast cannot be ruled out. However, a phylogenetic analysis that included virus, fungi, yeast, plant, *C. elegans* and *M. lignano* RdRPs strongly suggested that the *M. lignano* genes are coded by the nuclear genome (Additional file 5: Figure S4).

*M. lignano* has a basal place in the evolution of platyhelminthes [82]. The absence of RdRP genes in other flatworms may be due to the acquisition of other amplification mechanism or functional RdRP homologs. In humans, for example, the telomerase reverse transcriptase (TERT) show RdRP activity by using a snoRNA as RNA template, additionally, the dsRNAs produced by this TERT are processed by Dicer, and drive the silencing of the target RNA [83]. However, RdRPs family might display a high divergence at the protein sequence in platyhelminthes, not allowing their detection by blast search.

Additionally, we detected genes involved in the persistence of RNAi. This persistence depends on SMG genes 2-, 5- and 6-, possibly by facilitating the amplification of the RNAi signal [84] (Fig. 7a, Additional file 1: Table S7).

### Spreading of RNAi

In *C. elegans*, the cell-to-cell spread of the interfering signal has been reported, allowing a systemic silencing via endo/exo-RNAi pathways [55]. This phenomenon depends on SID-1 and the products of the RSD (from RNA spreading deficient) genes −2, −3 and −6. Mutants for RSD-2, RSD-3 and RSD-6 still retain the systemic RNAi in somatic cells, but are unable to further spread dsRNAs to the germline [85]. In *C.elegans* RSD-2, a protein with no predicted domains interacts with RSD-6, a Tudor domain containing protein. RSD-3 encodes an epsin N-terminal homology (ENTH) domain, suggesting that RSD-3 may play a role in the traffic of vesicles during systemic RNAi [85].

In flatworms, among all the factors of this pathway only homologous genes of RSD-3 were identified (Fig. 7b, Additional file 1: Table S8), suggesting that the RSD-2/RSD-6 system is either not conserved, or is too divergent to be detected by our method. Interestingly, similar results have been reported in insects [63], consistent with the observation that systemic RNAi is not possible in *D. melanogaster*. The demonstration of RNAi silencing by feeding in planaria [86] supports the existence of a functional spreading mechanism at least in this free living flatworm. No reports of similar feeding approaches have been reported so far in parasitic flatworms, so is not clear if spreading is limited to planaria or extended to all flatworms. In any case, efforts to unravel the players in RNAi spreading in planaria are warranted.

### Chromatin remodeling mediated by siRNA is possible

In addition to the cytoplasmic RNAi, in *C. elegans* mRNAs are silenced within the nucleus. NRDE-3, an Ago protein, binds to secondary siRNAs in the cytoplasm, transports them into the nucleus, and interacts with nascent transcripts in an siRNA-dependent manner [87]. We did not detected specific orthologues of NRDE-3 in flatworms; however, it is possible that the role of NRDE-3 is played by any of the other Agos detected in flatworms, including the flatworm-specific Agos. On the other hand, the absence of a canonical amplification pathway in all flatworms with the only exception of *M. lignano*, suggests that secondary siRNA might not be produced and consequently co-transcriptional silencing might not be operational in parasitic species.

It has been shown that RNAi also induces transcriptional gene silencing by modifying the chromatin structure [71]. If the targeted genes are expressed in the germline the silencing signal can be transmitted epigenetically to the next generations [71]. Interestingly here as well, a differential distribution was observed. Seven genes were conserved across flatworms, including GFL-1, the ATP-dependent DEAD/H box RNA helicase RHA-1, the metyl transferase PRMT-5, the heterochromatic protein HPL-1, the chromodomain containing protein MRG-1 and the polycomb complex proteins MES-2 and MES-6 (Fig. 7c, Additional file 1: Table S9). Only MES-3, a member of the polycomb complex, was not detected in flatworms, but it has been reported that MES-3 is a novel acquisition of nematodes and is not conserved in *D. melanogaster*, which only have functional homologs of MES-2 and MES-6 [88]. Remarkably, the zinc finger protein ZPF-1 was detected only in free living species and trematodes, being absent in cestodes. However, three paralogous genes of ZPF1 exist (PHF-14, PHF-15 and LIN-49 in Additional file 1: Table S9), being all of them present in flatworms, suggesting that the function might be conserved.

### Absence of a canonical PIWI pathway in parasitic flatworms

The methylase HEN-1, implicated in the siRNAs and piRNA associated to PIWI-like Argonautes [89] is only detected in *M. lignano* and *S. mediterranea* consistent with the exclusive presence of PIWI-like Agos in free living flatworms (Additional file 1: Table S9)*.*

The absence of Vasa, another key factor of piRNA pathway [40] and the lack of piRNA in small RNA sequencing data of parasitic platyhelminthes (Table 1) support the hypothesis of an evolutionary loss of piRNAs in parasitic flatworms. However, it has been reported that FL-Ago-2 and PL10 (a homolog of Vasa) transcripts are enriched in germline cells of *S. mansoni* [90], and as we mentioned above, FL-Ago-2 may be involved in TE silencing, suggesting that parasitic flatworms might have an alternative pathway to the PIWI pathway. In any case, other approaches as functional genomics analyses employing RNAi and transgenesis to define pathways and enzymes that participate in silencing of TEs and immunoprecipitations to characterize binding partners are needed to further decipher this enigma.

## Conclusions

Our data indicate that in all analyzed species most of the small RNA pathways are conserved, providing a strong bioinformatic evidence that gene-silencing mediated by RNAi and endogenous gene expression regulation mediated by small RNAs is possible in all the members of the phylum. While in parasitic flatworms there is a marked reduction in the repertoire of small RNA pathway factors, in *S. mediterranea* reduction is less pronounced and even lesser in *M. lignano* where a putative amplification pathway of siRNAs was identified (Fig. 8).

A general phenomenon of simplification has occurred in flatworms, especially in parasitic species [91–93]. We found that the losses of metabolic pathways are differential and related to the environment in the invaded tissue. In addition, a reduction in the set of flatworm miRNAs, greater in parasitic species compared to free-living organisms [23, 26], might suggest that the absence of miRNA families could be related to the loss of target mRNAs. This process of loss of redundancy in the biological system appears very early in the evolution of parasitic flatworms and is still unclear if it represents an adaptation to parasitism or is an ancestral characteristic shared between the Neodermata (as proposed by Hahn et al. [93]). However, it is tempting to hypothesize that, in the case of small RNAs, factors with similar function have been removed of the genomes of parasitic flatworms, while remaining factors could display multi-role in different pathways. This reduction in the small RNA machinery could affect the biogenesis of some small RNA, and could be related to the reported pronounced loss of miRNAs in the Neodermata species [23, 26].

On the other hand, we detected flatworm specific amplifications and variants. We found that Dcr-2 is structurally different in trematodes and that is duplicated in Opistorchidae and Fasciolidae. In addition, a flatworm specific group of argonaute proteins (FL-Agos) exist, being expanded independently in the diverse linages (Fig. 8). The relevance of these novelties are currently unknown, studies that combine both in silico and experimental approaches are needed to unravel their function.

Here we used a strict homology search method against genomes that are in most cases in a draft stage. We did not rely only in current annotation (since this might be partial or incorrect for many species) adding a whole genome homology search to confirm the putative absences. We are quite confident that this combined approach provide a good picture of the main aspects of small RNA pathways in flatworms. Other in silico approaches like PSI-BLAST search [94], a method for low-homology structural search [95] or transitive homology approaches [96] can be used in the future to search for more divergent sequences.

Additionally, experimental approaches are needed to confirm some of the results reported here and to detect novel factors that could be developed uniquely in flatworms. Functional genomics utilizing RNAi [5], transgenesis [97, 98], and more recently genome-editing by CRISPR-Cas9 [99] would help to define the function of some of the factors reported here, while immunoprecipitation assays could be used in the identification of novel factors [100].

In any case, we believe that the results reported here are relevant not only to shine a light on the basic biology and gene regulation mechanisms in these organisms, but also to optimize the RNAi as functional genomic tool for parasitic worms, agents of neglected tropical diseases affecting hundreds of million people worldwide.

## Methods

### Identification of small RNA pathways proteins and generation of local repository

Sixty-one proteins involved in small non-coding RNA pathways in *C. elegans* were identified from the literature (Additional file 1: Table S1), and the sequences were downloaded from wormbase (www.wormbase.org, release WS244). A local repository with the genomes and proteomes of 8 species of Trematodes, 12 species of Cestodes, 2 Monogeneans and 2 Turbelaria was generated (Table 1). The data was downloaded from Wormbase Parasite (http://parasite.wormbase.org) and The *Gyrodactylus salaris* Genome Project (http://invitro.titan.uio.no/gyrodactylus/). Results were later corroborated with the new genomic annotations published in Wormbase Parasite release 8.

### Homologous search with BLAST

The protein sequences of all *C. elegans* small RNA pathways factors were used to interrogate the proteomes of platyhelminthes using BLASTp [101]. All hits in the platyhelminthes proteomes with an expected value ≤1 E-05 were retained and reciprocally BLASTed (using the same e-value cut-off) to the *C. elegans* proteome. Only the best reciprocal hits between flatworms and *C. elegans* were retained for further inspection.

When no homologous protein was identified or the retrieved gene was incomplete or fragmented, a genomic search using tBLASTn was conducted. A careful inspection of the resulting High-scoring Segment Pairs (HSP) aligned to the genomes were useful to extend gene annotation or to fully annotate putative novel genes. These were confirmed by reverse tBLASTn to *C. elegans.* Alternatively, if a positive hit was retrieved in some flatworm species but not in others, these were used as queries for new genomic and proteomic reciprocal blast searches. This strategy helped to overcome the disparity on quality and completeness of genomic assemblies and/or gene annotation among the genomes deposited in Wormbase Parasite (http://parasite.wormbase.org/).

### Protein domains prediction

To confirm that the identified sequences from platyhelminthes were functional homologs of the *C. elegans* proteins, domains were predicted with HMMScan [102], using an expected value cut-off of 1 E-03. Only those sequences with a similar functional profile to the *C. elegans* protein were considered as orthologs. However, we consider flatworm genes to be homologs when reverse BLAST produced a hit to a paralogous of the gene of *C. elegans* involved in the small non-coding RNA pathways, to avoid confusion these cases are explicitly indicated in the results section. In cases where protein domains were not detected with HMMScan, MEME suite [103] was used to identify conserved amino acidic positions.

### Sequence alignment and phylogenetic tree building

Local alignment with MAFFT [104] was used to align the detected sequences in platyhelminthes with the *C. elegans* reference proteins and orthologous detected in other species. Alignments were visualized and edited when needed with BioEdit version 7.2.5 [105]. The alignments were used to build Maximum Likelihood (ML) phylogenetic trees with PhyML [106], with statistical branch support (SH-like). The model used to build the trees was inferred with Modelgenerator [107]. The trees were visualized with Evolview [108].

## Additional files

Additional file 1: Table S1. *C. elegans* small non-coding RNA pathway genes used for homology search. **Table S2.** Ribonuclease III genes identified in flatworms. **Table S3.** Argonaute genes identified in flatworms. **Table S4.** microRNA pathway genes detected in flatworms. **Table S5.** Exogenous RNA interference pathway genes detected in flatworms. **Table S6.** Endogenous RNA interference pathway genes detected in flatworms. **Table S7.** Amplification pathway genes detected in flatworms. **Table S8.** RNA interference spreading signal genes detected in flatworms. **Table S9.** Chromatin remodeling genes associated to RNA interference detected in flatworms. (XLSX 47 kb)

Additional file 2: Figure S1. Dcr-2 and Dcr-3 genomic location and expression in Fasciolidae and Opisthorchiidae. In *C. sinensis* and *O. viverrini* genomes both paralogues are separated by less than 10 kb, while, in *F. hepatica* the intergenic region is almost 50 kb. Transcriptomic data of *F. hepatica* show that both genes are transcribed in several developmental stages. (TIFF 1513 kb)

Additional file 3: Figure S2. FL-Agos genomic location and expression in *S. mansoni*, *F. hepatica* and *E. granulosus*. Transcriptomic data show that all FL-Agos are expressed in several developmental stages. Genes size was found to be variable ranging from around 4 kb in *E. granulosus* to 6.5 kb in *S. mansoni* and almost 40 kb in one of the *F. hepatica* genes. (TIFF 5708 kb)

Additional file 4: Figure S3. Motifs detected in putative GW182 sequences of flatworms. Motifs detected in UBA and RRM domains are indicated in purple and lightblue boxes, respectively. The GW182 family conserved motif of AGO binding domain was also found in all flatworms (yellow box). Two additional motifs conserved only among flatworms were detected. The motif at the AGO binding domain (white box) is conserved in all flatworms, while, the other (black box) is rich in glutamine residues (Q) and is only conserved in trematodes and cestodes. Sequences of common motifs to all flatworm linages were aligned and residue conservation is indicated. Additionally, the number of GW repeats for each sequence are indicated. Species with parcial or no predicted gene model are not shown (see Additional file 1: Table S3). (TIFF 3464 kb)

Additional file 5: Figure S4. A maximum likelihood tree of RNA dependent RNA Polymerases. One hundred iterations bootstrap was calculated. Values below 0.4 are not shown. (TIFF 1023 kb)

## Abbreviations

AGO: Argonaute family proteins
DCR: Dicer protein
dsRNA: double stranded RNA
endoRNAi: endogenous RNAi
ERI: Enhanced RNAi proteins
exoRNAi: exogenous RNAi
FL-AGO: Flatworm Specific Argonaute Proteins
miRISC: miRNA processing RISC complex
miRNA: microRNA
piRNA: Piwi protein interacting RNA
RdRP: RNA dependent RNA Polymerase
RISC: RNA Induced Silencing Complex
RNAi: RNA interference
siRISC: siRNA processing RISC complex
siRNA: small interfering RNA
