# Supplementary figures and images for: Conservation and diversification of small RNA pathways within flatworms
Source: BMC Evol Biol. 2017 Sep 11;17:215. doi: 10.1186/s12862-017-1061-5 (PMC5594548; doi:10.1186/s12862-017-1061-5)

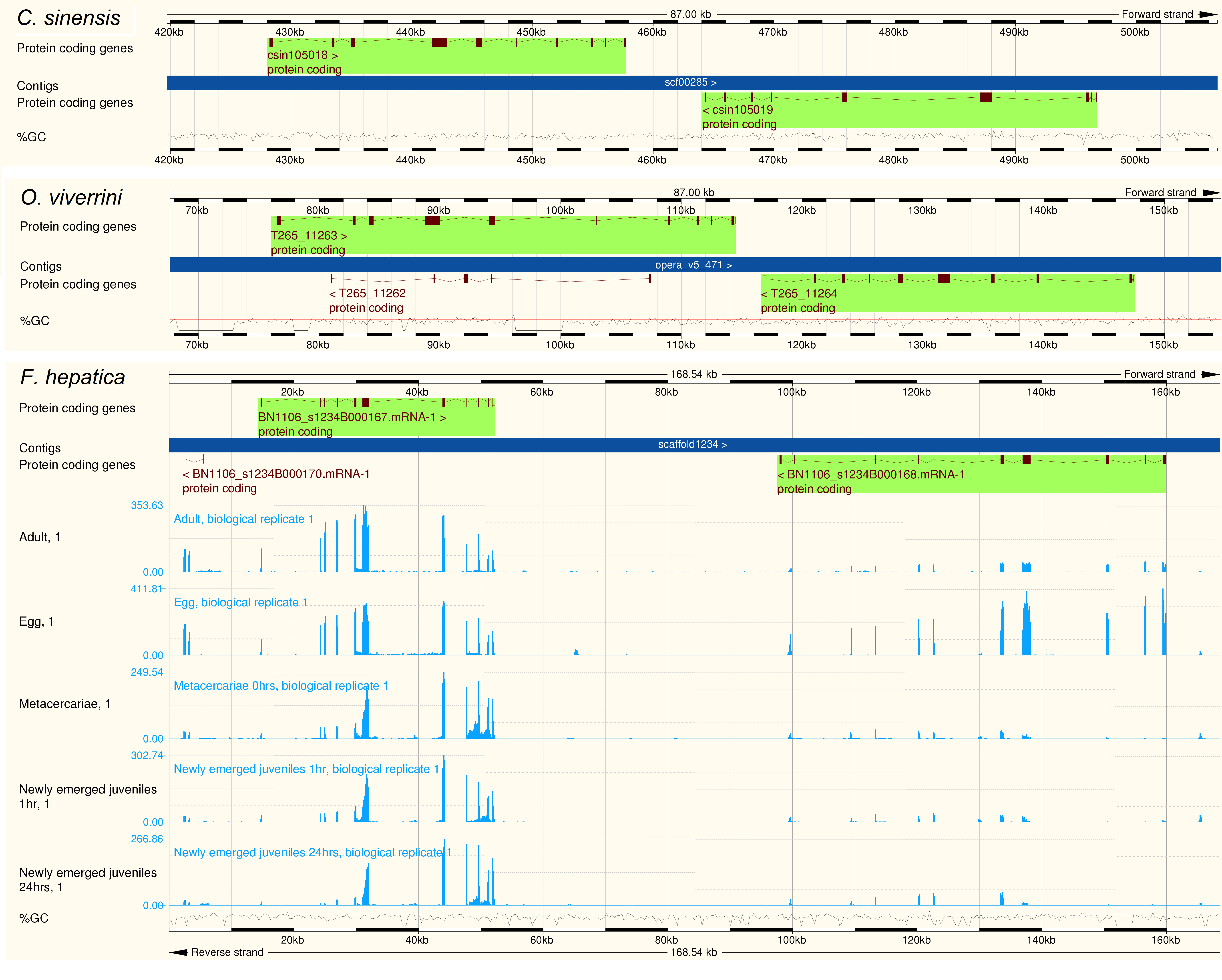

Supplement: Supplementary file 2 — Dcr-2 and Dcr-3 genomic location and expression in Fasciolidae and Opisthorchiidae. In C. sinensis and O. viverrini genomes both paralogues are separated by less than 10 kb, while, in F. hepatica the intergenic region is almost 50 kb. Transcriptomic data of F. hepatica show that both genes are transcribed in several developmental stages. (TIFF 1513 kb) [file 12862_2017_1061_MOESM2_ESM.tif]

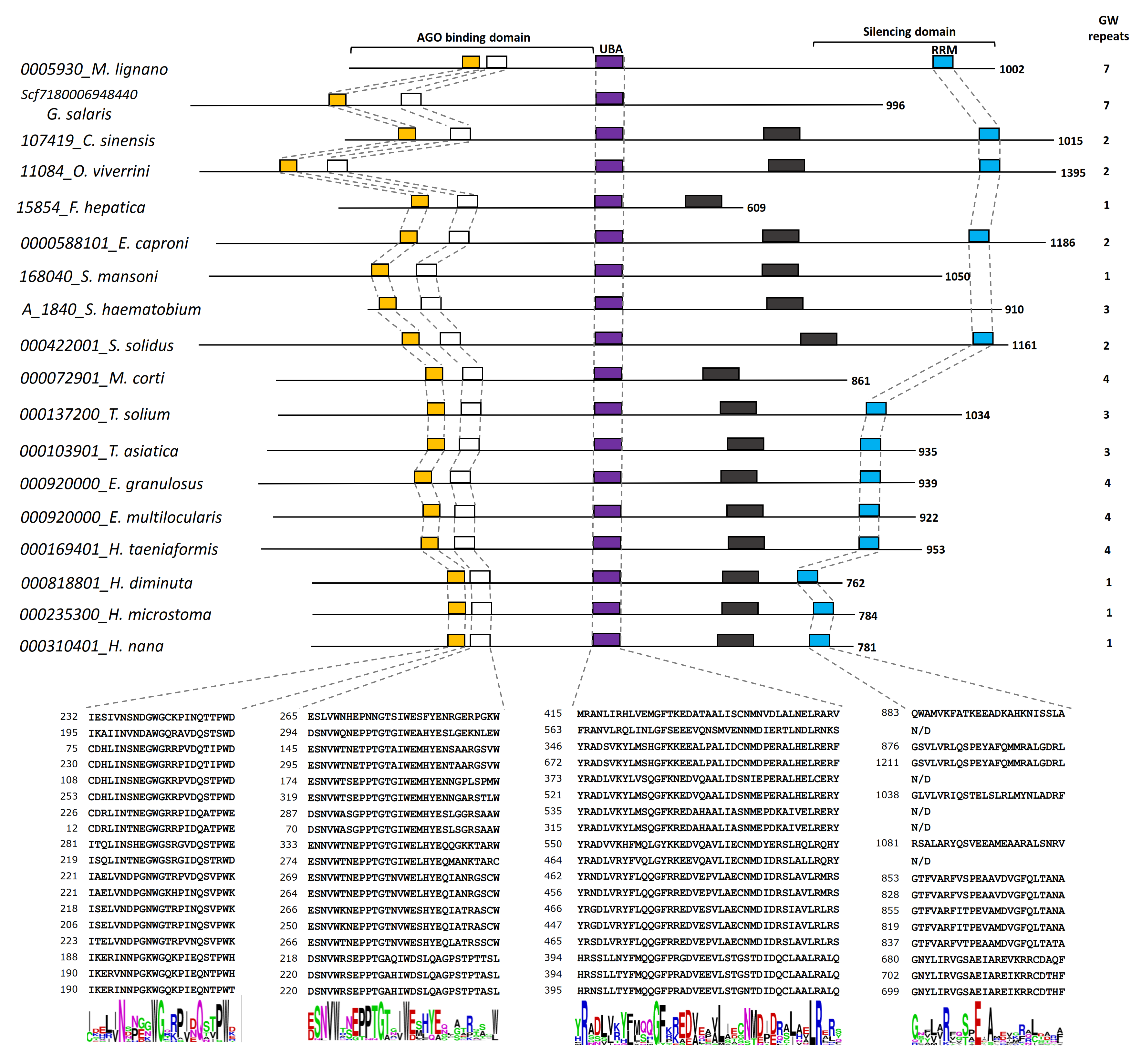

Supplement: Supplementary file 4 — Motifs detected in putative GW182 sequences of flatworms. Motifs detected in UBA and RRM domains are indicated in purple and lightblue boxes, respectively. The GW182 family conserved motif of AGO binding domain was also found in all flatworms (yellow box). Two additional motifs conserved only among flatworms were detected. The motif at the AGO binding domain (white box) is conserved in all flatworms, while, the other (black box) is rich in glutamine residues (Q) and is only conserved in trematodes and cestodes. Sequences of common motifs to all flatworm linages were aligned and residue conservation is indicated. Additionally, the number of GW repeats for each sequence are indicated. Species with parcial or no predicted gene model are not shown (see Additional file 1: Table S3). (TIFF 3464 kb) [file 12862_2017_1061_MOESM4_ESM.tif]

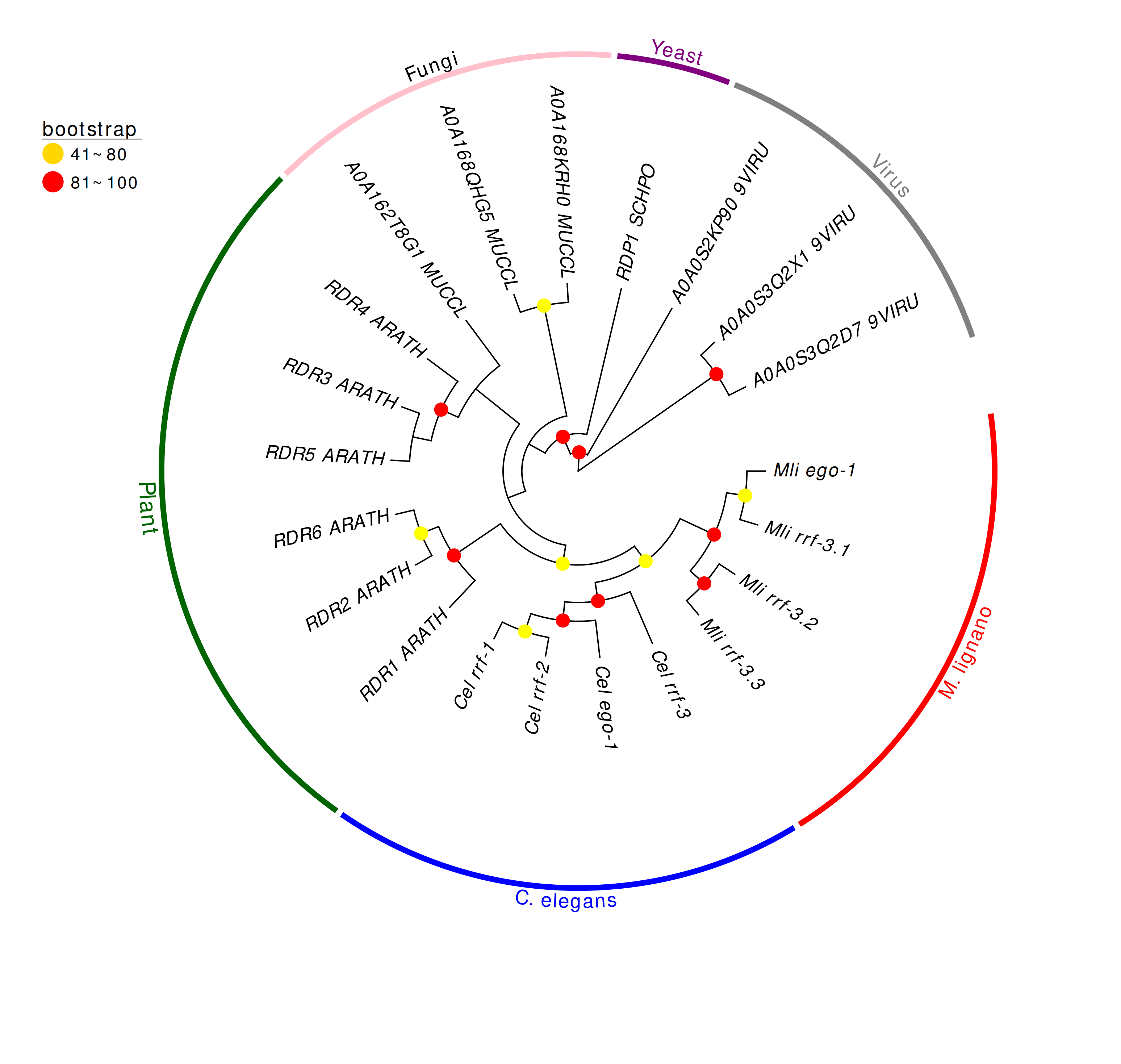

Supplement: Supplementary file 5 — A maximum likelihood tree of RNA dependent RNA Polymerases. One hundred iterations bootstrap was calculated. Values below 0.4 are not shown. (TIFF 1023 kb) [file 12862_2017_1061_MOESM5_ESM.tif]
